# Invasive mucinous adenocarcinoma of the lung revealed by a subcutaneous metastasis: A case report of a rare presentation

**DOI:** 10.1016/j.ijscr.2023.108964

**Published:** 2023-10-17

**Authors:** Anis Hasnaoui, Racem Trigui, Amal Benasr, Mariem Nouira, Fakhreddine Ben Abdallah

**Affiliations:** a Faculty of Medicine of Tunis, Tunis El Manar University, Rue Djebal Lakhdar 1006, Tunis, Tunisia; b Department of General Surgery, Menzel Bourguiba hospital, Tunisia; c Department of epidemiology, Menzel Bourguiba hospital, Tunisia; d Department of pathology, Menzel Bourguiba hospital, 7050 Menzel Bourguiba, Bizerta, Tunisia

**Keywords:** Secondary skin neoplasms, Non-small-cell lung carcinoma, Adenocarcinoma of lung, Biopsy, Case report

## Abstract

**Introduction:**

Lung cancer stands as the second most prevalent tumour and the foremost cause of cancer-related mortality. It typically manifests through respiratory symptoms. Subcutaneous metastases originating from pulmonary cancers are rare occurrences, with a remarkably low incidence.

**Presentation of case:**

A 53-year-old patient, with a history of smoking and unchecked chronic obstructive pulmonary disease, sought care at our outpatient clinic due to the rapid development of a subcutaneous mass. The patient underwent surgical excision of the mass under local anaesthesia. The anatomopathological examination confirmed the diagnosis of cutaneous metastasis from an infiltrative adenocarcinoma. A whole-body CT scan revealed an invasive mucinous adenocarcinoma of the lung. Consequently, palliative chemotherapy was initiated. Unfortunately, the patient succumbed to the disease three months later.

**Discussion:**

Subcutaneous metastasis originating from pulmonary sources is a rare phenomenon, with scant literature available on the subject. The presence of pulmonary cutaneous metastasis serves as an ominous sign of rapidly progressing and aggressive lung cancer. The prognosis in such cases is reserved, with an overall survival rate measured in months. The approach to management in these instances predominantly revolves around palliative chemotherapy, although surgical excision of metastases may be considered in select cases.

**Conclusion:**

Lung cancer unmasked by subcutaneous metastasis represents a rare clinical scenario. While chemotherapy remains the cornerstone of treatment, surgical intervention to remove metastases may be contemplated in a restricted subset of cases. Further research is imperative to ascertain the impact of surgery on both quality of life and overall survival.

## Highlights

• Skin metastases of lung cancer are very uncommon and represent evidence of the aggressive nature of the primary tumour.
• Invasive mucinous adenocarcinoma of the lung is a rare subtype of lung adenocarcinoma.
• Removal of skin metastases of lung cancer may hold promise in slightly improving patient outcomes.
• A rapidly evolving subcutaneous mass could potentially signal a malignant nature, necessitating thorough investigation.

## 1 Introduction

According to the most recent data from the Global Cancer Statistics 2020 report, lung cancer not only holds the distinction of being the second most prevalent form of cancer but also stands as the leading cause of cancer-related fatalities, claiming a staggering 1.8 million lives [1]. The 5-year overall survival rate for lung cancer is limited to 10–15 %, making it a disease with an extremely reserved prognosis [2]. Stage IV is characterized by the appearance of metastases, with the most common sites being the brain, bone, and liver. Subcutaneous locations are very uncommon and correlated with notably shorter overall survival [3]. Herein, we present a compelling case of bronchopulmonary cancer unveiled by a subcutaneous metastasis. This work has been reported in line with the SCARE criteria [4].

## 2 Presentation of case

**Fig. 1:**
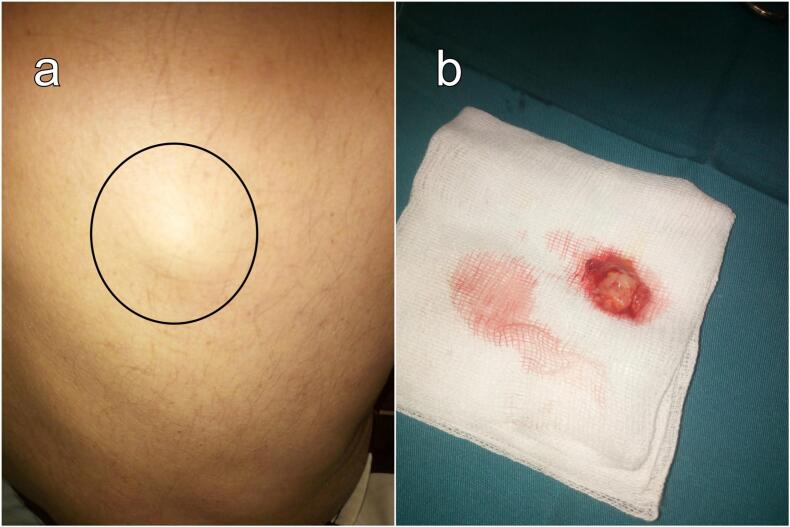
Subcutaneous metastasis. (a) Preoperative view: Subcutaneous nodule on the dorsum highlighted by a black circle. (b) Gross examination of the surgical specimen: Irregular, fibrous nodule measuring 22*14 mm surrounded by adipose tissue.

**Fig. 2:**
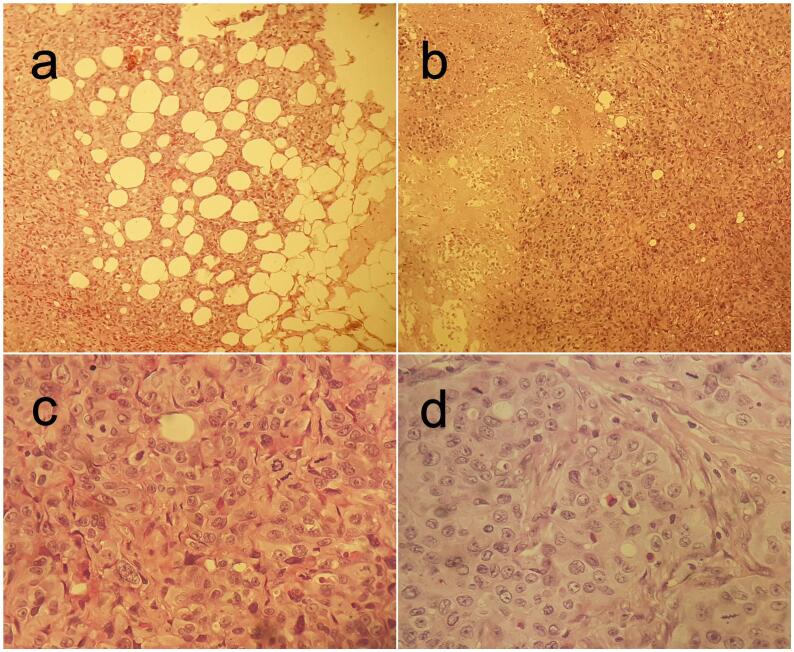
Histopathology findings. (a) Tumoral infiltration of subcutaneous fat x100. (b) Widely necrotic tumour proliferation x100. (c) Cellular details: nuclear enlargement, marked anisocytosis, numerous and abnormal mitoses x400. (d) Solid tumour architecture with glandular luminal formations x400.

**Fig. 3:**
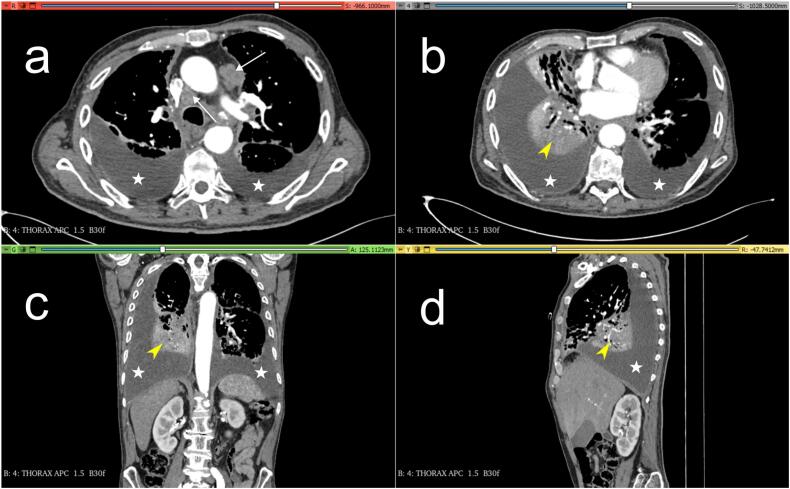
CT scan images. (a) and (b) axial views, (c) coronal view and (d) sagittal view, showing consolidation in the independent portion of the right lower lobe (Yellow arrow heads), bilateral pleural effusion (white stars), and lymphadenopathies (White arrows in (a)). (For interpretation of the references to colour in this figure legend, the reader is referred to the web version of this article.)

A 53-year-old patient, with a long history of smoking and unmonitored chronic obstructive pulmonary disease, was referred to our outpatient clinic for a rapidly evolving subcutaneous mass for a three-month duration. Upon examination, a distinctive, painless, and palpably solid subcutaneous nodule, measuring 2 by 2 cm, was discovered on the dorsum. Remarkably, there were no overt signs of local inflammation. The patient exhibited pursed-lip breathing, with a breathing rate of 22 cycles per minute. The oxygen saturation level was at 90 % and the auscultation revealed decreased breath sounds and dullness to percussion on both sides of the chest, indicative of bilateral pleural effusion. The patient underwent surgical full skin excision of the nodule down to subcutaneous fat under local anaesthesia and was referred to the pneumology department. Upon gross examination, the specimen revealed an irregular, fibrous nodule measuring 22 by 14 mm surrounded by adipose tissue (Fig. 1). The histopathological sections revealed an aspect of a metastasized location of an infiltrative adenocarcinoma with deposits of necrosis (Fig. 2). A whole-body CT scan revealed consolidation in the independent portion of the right lower lobe, bilateral pleural effusion along with thoracic lymphadenopathies, evoking a diffuse pneumonic-type lung adenocarcinoma (Fig. 3). Based on these findings, the diagnosis of an invasive mucinous adenocarcinoma of the lung was retained. Immunohistochemistry was deemed unnecessary. Consequently, palliative chemotherapy was initiated. Unfortunately, the patient succumbed to the disease three months later.

## 3 Discussion

Metastasis occurring in subcutaneous tissue is a rare occurrence, with an overall incidence rate of a mere 5.3 % [5]. The incidence of skin metastasis originating from lung cancer was 3.4 % in the metanalysis published by Krathen et al. [5]. In a substantial percentage of cases (approximately 20–60 %), it may manifest either preceding or concurrently with the diagnosis of lung cancer, warranting heightened concern, particularly in individuals with a history of smoking [6]. The ability of these metastatic cells to endure the inhospitable microenvironment of such unconventional sites serves as compelling evidence of the aggressive nature of the primary tumour, further underscoring our patient's grim prognosis [7]. Skin metastases resulting from pulmonary cancer most frequently manifest on the chest (28.4 %), abdomen (20.2 %), limbs (12 %), neck (11 %), back (11 %), scalp (7 %), pelvis (6 %), and face (5 %) [8].

The diagnostic challenge with skin metastasis arises from the diverse array of clinical presentations it can assume. These manifestations may take the form of fibrotic processes, nodules, cellulitis, zosteriform lesions, or ulcerations. Strikingly, these skin alterations can often be painless, displaying a spectrum of colours and sizes ranging from 2 mm to 6 cm [9,10]. Faced with this multifaceted clinical landscape, a myriad of differential diagnoses come into consideration, encompassing carcinomas (squamous and basal cells), melanomas, and carcinoid tumours [9]. While many experts concur that the prevailing clinical presentation often manifests as a rapidly proliferating, solid, and mobile nodule, it is worth noting that even this classic form may not conclusively point to a primary pulmonary origin [[11], [12], [13]]. Our patient presented with a rapidly advancing subcutaneous nodule. This accelerated progression should serve as a paramount factor for clinicians to weigh when assessing such lesions. It could potentially signal a malignant nature, necessitating thorough investigation and expedite diagnostic measures. The urgency in discerning the underlying pathology cannot be overstated, as it directly informs the subsequent course of action and treatment planning.

Malignancies in the upper lobes of the lungs exhibit a higher propensity for skin metastasis [14]. The majority of skin metastases tend to originate in proximity to the initial tumour, a phenomenon likely attributed to possible lymphatic dissemination. This pattern is further accentuated by the tendency of lung cancer to disseminate to regions of skin situated above the diaphragm [15]. Further, disparities in the prevalence of distinct histological types of lung cancer in the general population lead to variations in the propensity for skin metastasis. For instance, in some studies, adenocarcinoma emerges as the histological type most inclined to spread to the skin [16,17], while in others, large cell carcinomas assume this role with greater frequency [18,19]. In our case, the histological diagnosis was based on the examination of the subcutaneous metastasis showing an adenocarcinoma before identifying the origin of the metastasis in the lung. Based on the pathology report and CT images mimicking infectious pneumonia, we retained the diagnosis of an invasive mucinous adenocarcinoma of the lung. It is a rare and distinct subtype of lung adenocarcinoma [20], which adds to the peculiarity of our case.

In the traditional approach to stage IV lung cancer treatment, decisions are reached through multidisciplinary meetings. The cornerstone of treatment is palliative chemotherapy, contingent on the patient's overall health and performance status. Drug selection is tailored to the specific histological type. Since 2012, a new approach has emerged for stage IV patients with skin oligo-metastases. Those who underwent a combination of chemotherapy and surgical excision of skin metastases exhibited superior outcomes compared to patients receiving chemotherapy alone [7,21,22]. Even with the integration of chemotherapy and surgical intervention in highly selected cases, the prognosis for pulmonary cancer with skin metastasis remains reserved. The median survival, contingent on anatomopathological findings, ranges from 3 to 6 months [11]. In our case, the patient died three months after the initial diagnosis.

## 4 Conclusion

Skin metastases unveiling pulmonary cancer represent a seldom-discussed topic in medical literature, and their presence is typically indicative of a particularly reserved prognosis. While systemic treatment remains the cornerstone of management in this advanced stage, recent emerging research suggests that surgical removal of these metastases may hold promise in slightly improving patient outcomes. Clinicians should keep a high index of suspicion when encountering a rapidly evolving subcutaneous mass and not take the issue lightly because the prognosis may hinge upon a prompt diagnosis.

## Consent for publication

A written consent was obtained from the patient to publish this case report.

## Guarantor

Anis Hasnaoui.

## Registration of research studies

N/A.

## Ethical approval

Ethical approval was deemed unnecessary by our institutional ethical committee, as the paper is reporting a single case that emerged during normal practice.

## Funding

This research did not receive any specific grant from funding agencies in the public, commercial, or not-for-profit sectors.

## CRediT authorship contribution statement

Anis Hasnaoui: Conceptualization, Writing-Reviewing and Editing. Racem Trigui: writing-Original draft preparation. Amal Benasr: Data curation. Mariem Nouira: Writing-Reviewing. Fakhreddine Ben Abdallah: Data curation. All authors read and approved the final manuscript.

## Declaration of competing interest

The authors declare that they have no competing interests.
